# A systematic review and meta-analysis comparing mortality in pre-hospital tracheal intubation to emergency department intubation in trauma patients

**DOI:** 10.1186/s13054-017-1787-x

**Published:** 2017-07-31

**Authors:** Espen Fevang, Zane Perkins, David Lockey, Elisabeth Jeppesen, Hans Morten Lossius

**Affiliations:** 1 Department of Research and Development, Norwegian Air Ambulance Foundation, Drøbak, Norway; 2 Department of Anaesthesiology and Intensive Care, Stavanger University Hospital, Stavanger, Norway; 3 Blizard Institute, Centre for Trauma Sciences, Queen Mary University, London, UK; 4 London’s Air Ambulance, The Royal London Hospital, London, UK; 5 Department of Health Sciences, University of Stavanger, Stavanger, Norway

**Keywords:** Airway management, Intubation, Intratracheal, Trauma, Rapid sequence induction, Pre-hospital, Emergency medical services

## Abstract

**Background:**

Pre-hospital endotracheal intubation is frequently used for trauma patients in many emergency medical systems. Despite a wide range of publications in the field, it is debated whether the intervention is associated with a favourable outcome, when compared to more conservative airway measures.

**Methods:**

A systematic literature search was conducted to identify interventional and observational studies where the mortality rates of adult trauma patients undergoing pre-hospital endotracheal intubation were compared to those undergoing emergency department intubation.

**Results:**

Twenty-one studies examining 35,838 patients were included. The median mortality rate in patients undergoing pre-hospital intubation was 48% (range 8–94%), compared to 29% (range 6–67%) in patients undergoing intubation in the emergency department. Odds ratios were in favour of emergency department intubation both in crude and adjusted mortality, with 2.56 (95% CI: 2.06, 3.18) and 2.59 (95% CI: 1.97, 3.39), respectively. The overall quality of evidence is very low. Twelve of the twenty-one studies found a significantly higher mortality rate after pre-hospital intubation, seven found no significant differences, one found a positive effect, and for one study an analysis of the mortality rate was beyond the scope of the article.

**Conclusions:**

The rationale for wide and unspecific indications for pre-hospital intubation seems to lack support in the literature, despite several publications involving a relatively large number of patients. Pre-hospital intubation is a complex intervention where guidelines and research findings should be approached cautiously. The association between pre-hospital intubation and a higher mortality rate does not necessarily contradict the importance of the intervention, but it does call for a thorough investigation by clinicians and researchers into possible causes for this finding.

**Electronic supplementary material:**

The online version of this article (doi:10.1186/s13054-017-1787-x) contains supplementary material, which is available to authorized users.

## Background

Pre-hospital airway management is an important area for research in pre-hospital critical care [1]. Tracheal intubation (TI) with a correctly positioned cuffed tracheal tube is considered the gold standard for securing an airway [2–5]. Pre-hospital intubation (PHI) of trauma patients is performed in many advanced emergency medical systems (EMS). Alternatively, conservative airway measures may be used before hospital admission, with TI performed in the emergency department (ED) [6]. Outside the operating theatre and in out-of-hospital settings, TI is challenging, with relatively high complication rates and limited resources for managing complications [3, 7–11]. The reported success rates for PHI vary, but the best-performing systems show success rates similar to those of in-hospital emergency TI [12–16]. For patients not in cardiac arrest, emergency department intubation (EDI) is normally performed as rapid sequence induction intubation (RSI), which includes the use of a rapid-onset neuromuscular blocking agent before TI, whereas PHI is done both with and without drugs [17].

The indications, techniques and providers used for the procedure vary widely, and interpretations of the current evidence of the effects of PHI on patient outcome differ considerably [18]. Although several guidelines suggest that TI should be considered for all trauma patients with a Glasgow coma scale (GCS) score of 8 or below, the evidence supporting the use of a particular GCS score as a threshold for intubation is poor [2, 4, 5]. A 2009 Cochrane review of all types of emergency TI included three studies that fulfilled the Cochrane criteria and in which the majority of patients experienced out-of-hospital cardiac arrest. The authors’ conclusion regarding the subgroup of trauma patients in this analysis was that the current evidence base provided no imperative to expand the practice of pre-hospital intubation in urban systems [19]. This systematic review was performed to compare the mortality rates of adult trauma patients undergoing PHI to those undergoing EDI.

## Methods

### Protocol and registration

The study was registered in the PROSPERO database in July 2014 under registration number CRD42014012968 and is reported in accordance with the Preferred reporting items for systematic reviews and meta-analyses (PRISMA) guidelines [20].

### Eligibility criteria

All full-text original articles comparing the mortality rates of adult trauma patients who received PHI to those treated with basic airway management and subsequent EDI were considered. Only articles published in English were included in the search.

### Exclusion criteria

Review articles, conference and meeting abstracts, letters and editorials were excluded. Publications that did not specify PHI or EDI for all patients, and those investigating paediatric patients, burn patients and patients with medical conditions, including cardiac arrest, were excluded. Studies considered by our assessment table to be of poor quality were excluded from the meta-analyses.

### Search

In co-operation with a librarian, we searched the following databases: EMBASE (1974 to 11 July 2016), MEDLINE (1946 to 11 July 2016) and the Cochrane Library (up to 11 July 2016). All word variations and thesaurus terms connected to “pre-hospital” and “emergency medicine systems” in the respective search engines were combined with the word variations and thesaurus terms of “intubation” and “airway management”. Reference lists of electronically identified publications, including review articles, were screened for studies that were not identified by the initial data search. When outcome data were missing or unclear, we attempted to contact the authors directly by email. See Additional file 1 for the full search strategy.

### Study selection

Two reviewers (EF and ZP) independently screened the titles and abstracts of all records identified in the searches. Disagreements were resolved via discussion. A data extraction form that included study design, provider type, patient category and outcome data was developed.

### Assessment of study quality and risk of bias in the included studies

In accordance with the Cochrane principles and the Grading of recommendations assessment, development, and evaluation (GRADE) approach, risk of bias in randomized trials was assessed as high, low or unclear for allocation concealment, blinding, incomplete outcome data, selective reporting and other limitations [21, 22]. Randomized trials are considered by the GRADE approach to provide high-quality evidence in the absence of important limitations. For observational studies, an assessment table was developed based on the principles stated by the MOOSE group and the National Institutes of Health (Additional file 2) [23, 24]. Each observational study was examined for clear definitions of the study population, clear definitions of outcomes and outcome assessment in both of the patient groups, directly comparable patient groups, consistent results, identification of important confounders and prognostic factors and the absence of serious methodological limitations. The methodological quality of the individual observational studies was rated as good, fair or poor. In the GRADE approach, observational trials without special strengths or important limitations are considered to provide low-quality evidence.

### Data items and statistical analysis

Odds ratios (OR) and adjusted odds ratios (AOR) for mortality and details of the study methodology, patient population (all trauma or traumatic brain injury (TBI) only), whether the service provided RSI for all pre-hospital patients, whether the study was set in a mainly physician-manned EMS (like some European services) or paramedic-manned EMS (like most American services) and whether physicians treated all patients who underwent PHI were extracted. Clinical data on median year of inclusion, injury severity score (ISS), GCS, percentage of patients in shock, systolic blood pressure and follow-up time were also extracted. The authors’ main conclusions on the impact of PHI on mortality rates were registered as favourable, unfavourable, inconclusive or no proven difference.

Odds ratios (OR) were analysed with the Mantel-Haenszel method using the analysis model for random effects. A random effect model was chosen over a fixed effect model as the impact of the intervention on the mortality rate may differ considerably between patient groups. As a wide range of different patient groups were predicted to be represented in the full search, the true effects for the studies were likely to vary, and a random effect model was considered to give a more valid result. Analyses of AOR were performed using the generic inverse variance model for random effects for dichotomous data. We calculated pooled odds ratios and 95% confidence intervals (CI) where appropriate.

All statistical analyses were performed using the Review Manager programme [25]. Forest plots were constructed for unadjusted and adjusted mortality, subdivided into studies in which all patients in the PHI group received RSI and studies where none or only some of the patients in the PHI group received RSI.

### Additional analyses

To reduce the impact of known possible sources of heterogeneity and to determine whether data from the same material could yield a different result if examined in a different setting, data from the initial mortality analysis were subdivided for three additional analyses: studies with no significant differences in ISS, studies with a comparable GCS score <9 and studies in which most PHIs were performed by physicians.

A table was created for the summary of findings according to the GRADE methodology [26]. Forest plot analyses were conducted to compare the mortality rates for PHI and EDI across studies. The possibility of publication bias was examined using funnel plots for unadjusted and adjusted mortality.

## Results

**Fig. 1:**
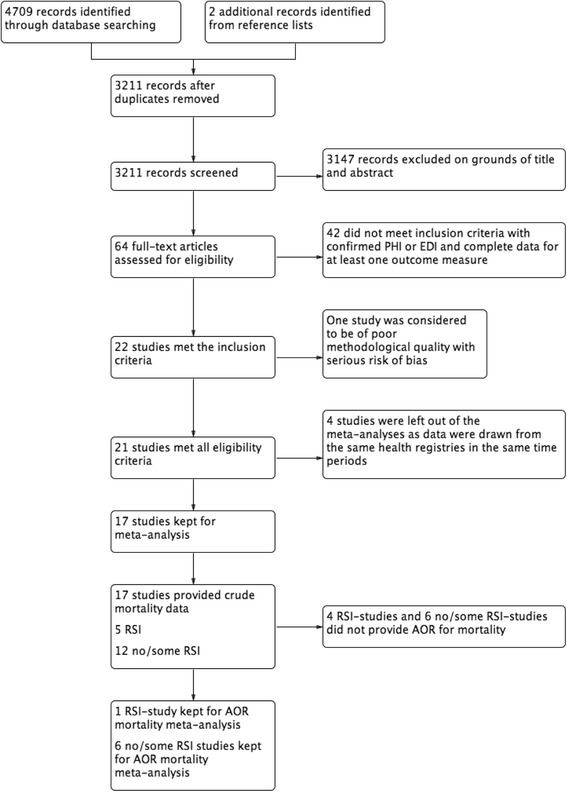
Study flow diagram. *PHI* pre-hospital intubation, *EDI* emergency department intubation, *RSI* rapid sequence induction intubation, *AOR* adjusted odds ratio

The search identified 3211 unique references through the search process described in Fig. 1. After the initial screening of titles and abstracts of all records, 64 studies were examined in full text by both authors responsible for the selection process. Of the 64 studies, 42 were excluded because PHI or EDI was not confirmed for all patients. Twenty-two studies met our inclusion criteria and compared mortality rates of patients who underwent PHI with patients who underwent EDI (Table 1) [16, 27–47]. One study was considered to have poor methodological quality and was excluded. Seven studies that met the inclusion criteria reported data from the same health registries during the same period; of these, the three that best agreed with our defined aims were included in the meta-analysis, the others were excluded (Table 3). Two studies investigated different subgroups from a large trauma registry and both were included in the meta-analyses. One randomized controlled trial (RCT) and sixteen observational studies were included in the mortality meta-analysis. Five of the seventeen studies examined pre-hospital RSI. One RSI study and six of the twelve studies involving no RSI or some RSI provided adjusted data in their analyses (Table 2). Data from the primary analysis were used to perform separate subgroup analyses of four studies with no significant differences between groups in the ISS and four studies with a verified similar pre-hospital GCS score <9 in both groups (Table 3).

**Table 1:** Overview of included studies

Study ID	Type of study	Date	Nation (main)	All patients treated by physicians	Patients	ISS similar between groups	RSI for all patients	Verified intubation in ED of control group patients	Type of mortality measure	Exclusion of patients dying in the pre-hospital or ED phase	Study size
Al-Thani 2014 [27]	Retrospective database study	2010–2011	Qatar	No	Trauma	No	Yes	Yes	Not specified	Patients who died on scene before ETI excluded	311
Arbabi 2004 [28]	Retrospective database study	1994–2001	USA	No	Trauma	No	Medications given, not specified	Yes	Not specified	Not specified	4317
Bernard 2010 [29]	Randomized Controlled Trial	2004–2008	Australia	No	TBI	Yes	Yes	Yes	In-hospital	No	312
Bochicchio 2003 [30]	Observational Cohort study	2000–2001	USA	No	TBI	Yes	Yes	Yes	Not specified	Yes, within 48 hours (because of nonsalvageable TBI or donors)	191
Bukur 2011 [31]	Retrospective database study	2005–2009	USA	No	TBI	No	No	Yes	Not specified	DOA, in the pre-hospital environment, died in the ED, or any AIS = 6 excluded	2366
Davis 2005 (I) [33]	Retrospective database study	1987–2003	USA	No	TBI	No	No, only for some patients	Yes	Not specified	Death in the field or <30 minutes after ED admission excluded	4247
Davis 2005 (II) [32]	Retrospective database study	1987–2003	USA	No	TBI	No	No, only for some patients	Yes	Not specified	Death in the field or <30 minutes after ED admission excluded	2243
Eckert 2004 [35]	Retrospective database study	1998–2002	USA	No	Trauma	No	No	Yes	Not specified	Yes, up to 48 hours	244
Eckert 2006 [34]	Retrospective database study	1994–2003	USA	No	Trauma	No	No	Yes	Not specified	Yes, up to 24 hours	415
Eckstein 2000 [36]	Retrospective review of medical records	1993–1995	USA	No	Trauma	No	No	Yes	In-hospital	Some confirmed deaths in the ED included	496
Evans 2010 [38]	Retrospective database study	2007–2008	USA	No	Trauma	Yes	Yes	Yes	In-hospital	Death or discharge within 48 hours excluded	572
Evans 2013 [37]	Retrospective database study	2002–2009	Canada	No	TBI (trauma + GCS <9)	No	No	Yes	In-hospital	Patients that received pre- or in-hospital CPR excluded from mortality analysis	1027
Franschman 2011 [39]	Retrospective database study	2003–2007	the Netherlands	No	TBI	No	No, only for some patients	Yes	In-hospital	Not specified, but only patients with a CT-confirmed TBI included.	335
Irvin 2010 [40]	Retrospective database study	2000− 2005	USA	No	TBI (trauma + GCS 3)	No	No	Yes	In-hospital	Only patients with circulation at hospital admission included	8748
Oswalt 1992 [41]	Retrospective database study	1988–1989	USA	No	Trauma	No	No	Yes	Not specified	Deaths during resuscitation in the ED excluded	44
Shafi 2005 [42]	Retrospective database study	1994–2002	USA	No	Hypovolemic TBI (trauma + GCS <9)	No	No	Yes	In-hospital	ED deaths included Difference in mortality persisted in analysis of mortality for patients that survived beyond the ED	8786
Sloane 2000 [17]	Retrospective database study	1988–1995	USA	No	Trauma	Yes	Yes	Yes	30 days	Not specified	75
Sollid 2010 [43]	Retrospective review of medical records	1994–2005	Norway	Yes, anaesthesiologists	Trauma	No	Yes	Yes	In-hospital	No	287
Tracy 2006 [44]	Retrospective database study	2002–2003	USA	No	Trauma	No	No information	Yes	Not specified	Yes, up to 48 hours	628
Tuma 2014 [45]	Retrospective database study	2008–2011	Qatar	No	TBI (head AIS ≥3 and GCS <9)	Yes	Yes	Yes	30 days	Yes, up to 24 hours	160
Vandromme 2011 [46]	Prospective cohort study	2006–2009	USA	No	TBI	No	No, only for some patients	Yes	Not specified	Not specified, but only patients with a CT-confirmed TBI included	149
Wang 2004 [47]	Retrospective database study	2000–2002	USA	No	TBI	No	No, only for some patients	Yes	In-hospital	No, deaths in the ED included	4098

*ISS* injury severity score, *RSI* Rapid sequence induction, *ED* emergency department, *TBI* Traumatic brain injury, *GCS* Glasgow coma scale, *DOA* dead on arrival, *CPR* cardiopulmonary resuscitation, *CT* computed tomography * AIS* abbreviated injury scale, *ETI *endotracheal intubation

**Table 2:** Clinical information

Study ID	Mortality PHI	Mortality EDI	ISS PHI	ISS EDI	GCS PHI	GCS EDI	Percentage in shock/average SBP PHI	Percentage in shock/average SBP EDI	AOR PHI vs. EDI	Methodological quality determined by assessment tool	Conclusion of article
Al-Thani 2014 [27]	126/239	45/243	25.3	21.3	6.9	12.1	avg 127.9	avg. 129.4	AOR 2.4 (0.61–9.44) GCS, ISS, head injury	Fair	PHI associated with worse outcome
Arbabi 2004 [28]			Not specified	Not specified					AOR in favour of PHI 3.0 (1.9–4.9). Compared to patients not intubated at all, AOR 1.1 (0.7–1.9)	Poor	PHI associated with better outcome, but analysis included GCS in paralyzed patients
Bernard 2010 [29]	53/160	55/152	30.5	30.1	5	5	avg 128	avg 129	Randomized patients, no AOR given	Good	PHI had no significant impact on mortality, but improved neurological outcome
Bochicchio 2003 [30]	18/78	14/113	20.1	19.2	4	4.4	avg 105	avg 111	No adjustments made, but no significant differences between groups	Fair	PHI associated with worse outcome
Bukur 2011 [31]	55/61	286/2305	26.7	18.4	3.3	11.7	73.8	4.5	5 (1.7–13.7), adjusted for mechanism of injury, mean SBP, hypotension, mean GCS, GCS <8, head AIS, mean ISS, ISS >16	Fair	PHI associated with worse outcome
Davis 2005 (I) [33]	1390/2414	537/1833	36.6	28.3	4.4	8	72	50	AOR 2.12 (1.81–2.5). AOR in article given as an inverse variant (0.47 (0.40–0.55). Corrected for age, gender, mechanism of injury, GCS, head AIS, ISS, shock	Fair	PHI associated with worse outcome
Davis 2005 (II) [32]	531/1250	428/993							AOR 1.42 (1.13–1.78) adjusted for age, sex, mechanism, preadmission hypotension, head AIS, ISS and pre-intubation GCS	Fair	PHI by aeromedical teams associated with better outcome than EDI after ground transport
Eckert 2004 [35]			26	18	4	8	avg 132	avg 132	No AOR given	Fair	PHI associated with worse outcome
Eckert 2006 [34]	16/62	51/353	21	19	5	7	avg 104	avg 125	AOR for intubation in the field 2.3 (1.1-4.9), ED 3.6 (2.5-5.2), inpatient 0.28 (0.2–0.4)	Fair	PHI associated with worse outcome
Eckstein 2000 [36]	87/93	268/403	35	29					AOR for pre-hospital intubation 5.3 (2.3–14.2). Corrected for sex, mechanism of injury and ISS	Fair	PHI associated with worse outcome
Evans 2010 [38]	32/412	10/160	27.2	27	4.1	11.6	avg 122,4	avg 125,5	No AOR given	Fair	No significant differences in outcomes between groups
Evans 2013 [37]	182/269	315/758	31	26	3	6	28.8	15.3	Logistic regression analysis including hypotension, age, ISS, GCS. Pre-hospital intubation 2.8 (1.1–7.6) Trauma centre intubation 2.6 (1.3–5.6)	Fair	PHI associated with worse outcome
Franschman 2011 [39]	101/233	42/103	32	25	3	5	23	11	No AOR given	Fair	No significant differences in outcomes between groups
Irvin 2010 [40]	1539/2491	2985/8457	31.6	24.2	3	3	avg 121,3	avg 130,1	Corrected for ISS, SBP, penetrating or blunt trauma, age, head injuries and improved GCS en route. For all patients AOR 1.93 (1.74–2.15) Head injury only AOR 1.99 (1.35–2.93) Body injury only AOR 2.54 (1.85–3.48)	Fair	PHI associated with worse outcome
Oswalt 1992 [41]	9/18	9/26	31.4	24.7	5.2	5.9	avg 78,5	avg 131	No AOR given	Fair	No significant differences in outcomes between groups
Shafi 2005 [42]	818/1185	4105/7601	35	33	3.7	4.1	48	33	Logistic regression including age, ISS, specific injuries, pre-existing conditions, PH-fluids and CPR. Survival 0.531 (0.441–0.65) Inverse value: 1.88 (1.54–2.32)	Fair	PHI associated with worse outcome
Sloane 2000 [17]	3/21	12/54	31.4	29	5.2	5.8			No AOR given	Fair	No significant differences in mortality between groups, but higher occurrence of pneumonia in PHI group
Sollid 2010 [43]	108/240	10/47			3	6			No AOR given	Fair	Beyond scope of article
Tracy 2006 [44]	86/271	101/357	25.3	22.4	4	8.3			No AOR given	Fair	No significant differences in outcomes between groups
Tuma 2014 [45]	57/105	17/105	28	27			avg 129	avg 142	Unclear rationale for AOR	Fair	PHI associated with worse outcome
Vandromme 2011 [46]	30/64	35/85	38	33.7	4.1	5.9	avg 127,4	avg 151,3	Adjusted for GCS, SBP, RR and ISS. Adjusted RR 0.68 (0.36–1.19). Not possible to work out adjusted odds ratio	Fair	No significant differences in outcomes between groups
Wang 2004 [47]	871/1797	649/2301					21.8	8.7	Multivariate logistic regression with ISS, AIS-head and admission SBP. AOR 3.99 (3.21–4.93)	Fair	PHI associated with worse outcome

*PHI* pre-hospital intubation, *EDI* emergency department intubation, *ISS* injury severity score, *GCS* Glasgow coma scale, *SBP* systolic blood pressure, *avg* average, *AOR* adjusted odds ratio, *ED* emergency department, *RR* risk ratio *﻿, A﻿IS* abbreviated injury scale

**Table 3:** Inclusion overview

Study ID	Included in crude data mortality analysis RSI	Included in crude data mortality analysis non-RSI/not all RSI	Included in adjusted mortality analysis, RSI	Included in adjusted mortality analysis no/some RSI	Included in mortality analysis, no difference in ISS	Included in mortality analysis, comparable GCS scores below 9	Reason for exclusion
Al-Thani 2014 [27]	Yes		Yes				
Arbabi 2004 [28]							Did not meet assessment criteria
Bernard 2010 [29]	Yes				Yes	Yes	
Bochicchio 2003 [30]	Yes				Yes	Yes	
Bukur 2011 [31]		Yes		Yes			
Davis 2005 (I) [33]		Yes		Yes			
Davis 2005 (II) [32]							Conflict with Davis 2005(I)
Eckert 2004 [35]							Conflict with Eckert 2006
Eckert 2006 [34]		Yes					
Eckstein 2000 [36]		Yes		Yes			
Evans 2010 [38]	Yes				Yes		
Evans 2013 [37]		Yes					
Franschman 2011 [39]		Yes					
Irvin 2010 [40]		Yes		Yes		Yes	
Oswalt 1992 [41]		Yes					
Shafi 2005 [42]		Yes		Yes		Yes	
Sloane 2000 [17]							Conflict with Davis 2005(I)
Sollid 2010 [43]	Yes						
Tracy 2006 [44]		Yes					
Tuma 2014 [45]					Yes		Conflict with Al-Thani 2014
Vandromme 2011 [46]		Yes					
Wang 2004 [47]		Yes		Yes			

*RSI* rapid sequence induction, *ISS* injury severity score, *GCS* Glasgow coma scale

Of the 21 studies that met all the eligibility criteria, twelve concluded that PHI was associated with a worse outcome than EDI, seven found no differences in mortality between the groups, one found a lower mortality rate when PHI performed by aeromedical crews was compared with EDI provided after ground transport, and for one study﻿, a mortality analysis was beyond the scope of the article.

### Results of included studies

The clinical information, outcome data, quality assessment findings, results and main conclusions are shown in Table 2. Table 3 shows which studies were included in the different meta-analyses.

### Mortality meta-analysis

**Fig. 2:**
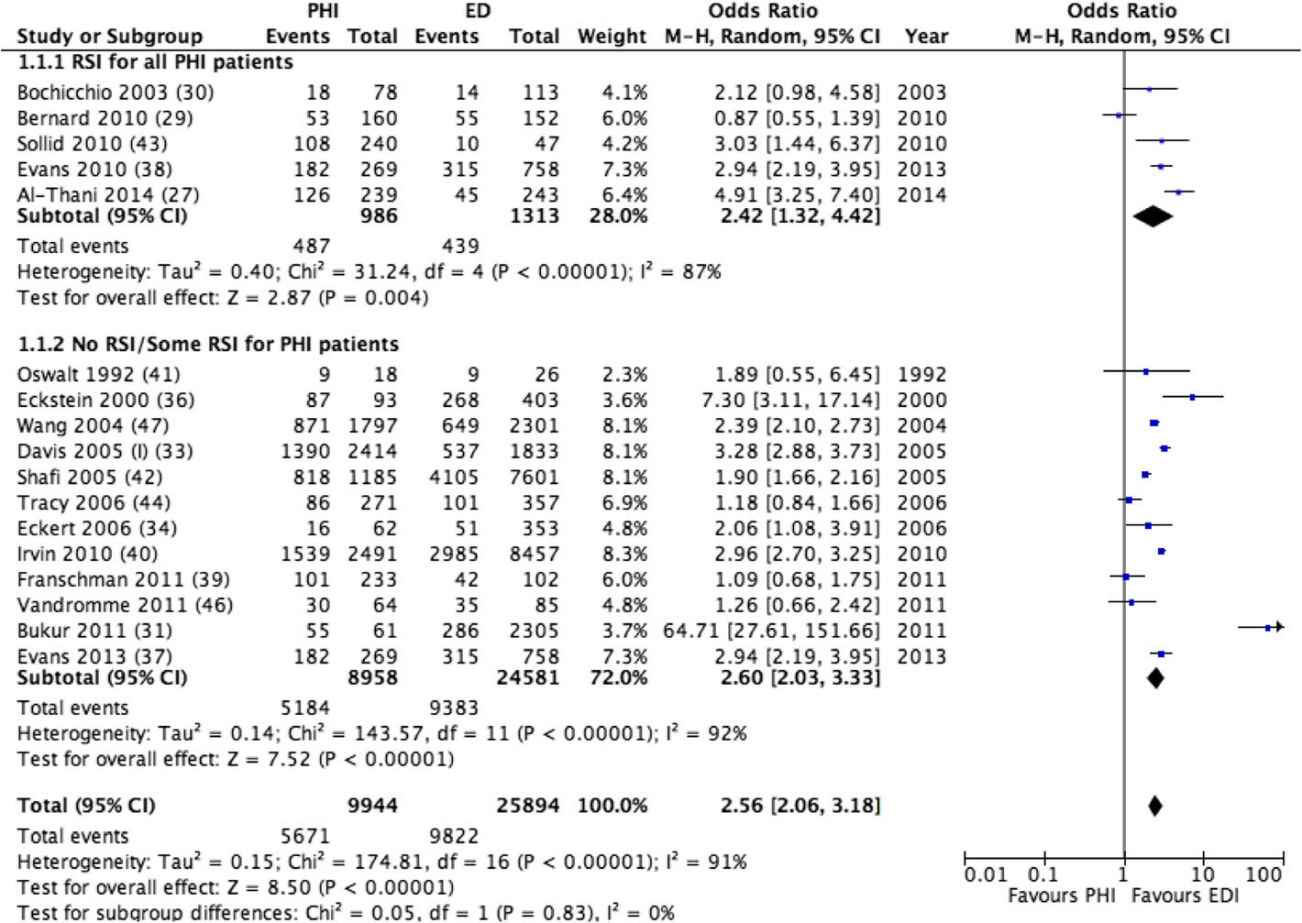
Mortality rates in pre-hospital intubation (*PHI*) versus emergency department intubation (*EDI*). *RSI* rapid sequence induction, *M-H* Mantel Haenszel

Seventeen studies investigating 35,838 patients were included in the mortality meta-analysis. The median mortality rate was 48% (range 8–94%) for PHI and 29% (range 6–67%) for EDI. A comparison using the Mantel-Haentszel method for random effects yielded an OR with 95% CI of 2.56 (2.06, 3.18) in favour of EDI. The forest plot was divided into two parts: one where all the patients in the PHI group had access to RSI and one where none or only some of the patients in the PHI group had access to RSI. When analysed separately, both comparisons were in favour of EDI. The OR was 2.42 (1.32, 4.42) for the RSI group and 2.60 (2.03, 3.33) for the no RSI/some RSI group (Fig. 2).

**Fig. 3:**
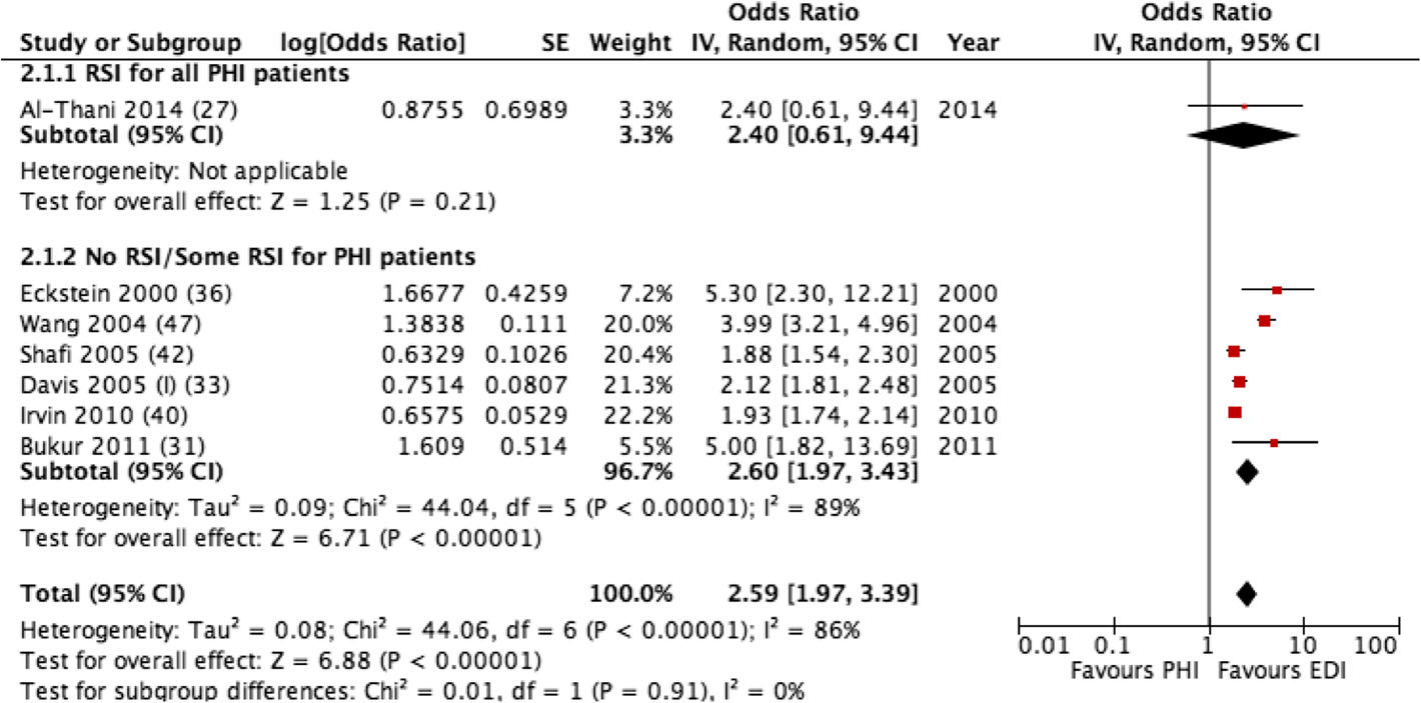
Adjusted odds ratios for mortality rates of pre-hospital intubation (*PHI*) versus emergency department intubation (*EDI*). *RSI* rapid sequence induction

Most studies included information on the clinical parameters associated with injury severity and used some form of correction before drawing a conclusion about the effect. The statistical rationale behind this decision varied among the studies, and AOR were provided in seven studies. Although the adjustment factors varied among the studies, all included adjustments for the ISS, five included adjustments for head injury and four included adjustments for blood pressure parameters (Table 2). When examining the seven studies that provided AORs, there was a trend in favour of EDI in all of them, with an AOR of 2.59 (1.97, 3.39). Viewed separately, the only RSI study had a mortality rate AOR of 2.40 (0.61, 9.44); the no RSI/some RSI group had an AOR of 2.60 (1.97, 3.43) (Fig. 3).

Four studies with a total of 1690 patients observed no significant differences between groups in the ISS, and all provided RSI for PHI patients. These studies showed a significantly higher mortality rate in the PHI group, with an OR of 1.94 (1.02, 3.70).

Four studies included patients with a GCS score <9 and no significant difference between the two groups in the scores. Two of these were RSI studies and the other two did not provide RSI for all PHI patients. There were no significant differences in the mortality OR in the RSI group (1.29 (0.54, 3.05)), but a significantly higher OR for mortality was found in the no RSI/some RSI group (2.40 (1.52, 3.77)).

Two studies were set in a European-organized EMS, where physicians perform most PHI [39, 43]. One of these studies included some paramedic-performed PHI without drugs. A subgroup analysis showed no significant differences in mortality rate between the groups, with an OR of 1.74 (0.64, 4.73).

We aimed to perform a subgroup analysis of studies in which trained physicians treated all patients in the PHI group, to determine if a similar level of experience with TI in both groups would affect the outcome. Only one such study was included, this was an observational study of anaesthesiologists, in which mortality was not a primary outcome. No correction for injury severity was attempted, yielding an OR for lower mortality in the EDI group of 3.02 (1.44, 6.37).

Forest plots of subgroup analyses can be found in Additional file 3.

A table for a summary of findings was developed in accordance with the GRADE methodology and is shown in Table 4.

**Table 4:** Summary of findings

Prehospital intubation compared to emergency department intubation for unconscious trauma patients:					
Outcomes	Number of participants (studies) Follow up	Quality of the evidence (GRADE)	Relative effect (95% CI)	Anticipated absolute effects*	
				Risk with emergency department intubation	Risk difference with prehospital intubation
Morality (RSI)	2299 (5 observational studies)	Very low^a, b, c^	OR 2.42 (1.32 to 4.42)	334 per 1 000	214 more per 1000 (64 more to 355 more)
Mortality (no RSI/some RSI)	33,539 (12 observational studies)	Very low^a, b, d, e^	OR 2.60 (2.03 to 3.33)	382 per 1 000	234 more per 1000 (174 more to 291 more)
Mortality, GCS similar and <8 (RSI)	503 (2 observational studies)	Very low^a, b, c^	OR 1.11 (0.75 to 1.65)	260 per 1 000	21 more per 1000 (51 fewer to 107 more)
Mortality, GCS similar and <8 (no RSI/some RSI)	19,824 (2 observational studies)	Very low^a, b, d^	OR 2.57 (2.38 to 2.77)	439 per 1 000	229 more per 1000 (212 more to 245 more)
Patients with no difference in injury severity	1690 (4 observational studies)	Very low^a, b, c^	OR 1.94 (1.02 to 3.70)	372 per 1 000	163 more per 1000 (5 more to 315 more)

*CI* confidence interval, *OR* odds ratio, *RSI* rapid sequence induction, *GCS* Glasgow coma score. *GRADE* Working Group grades of evidence: high quality **-** we are very confident that the true effect lies close to that of the estimate of the effect; moderate quality **-** we are moderately confident in the effect estimate: the true effect is likely to be close the estimate of the effect, but there is a possibility that it is substantially different; low quality **-** our confidence in the effect estimate is limited: the true effect may be substantially different from the estimate of the effect; very low quality **-** we have very little confidence in the effect estimate: the true effect is likely to be substantially different from the estimate of effect

^a^Observational studies

^b^High *I* squared score implies uncertain effect estimate, but most studies have overlapping CI

^c^The only source of high-quality evidence includes no effect, in contrast to the remaining studies

^d^Widely defined patient populations across studies

^e^Optimal size criterion met and combined 95% CI excludes no effect

*The risk in the intervention group (and its 95% CI) is based on the assumed risk in the comparison group and the relative effect of the intervention (and its 95% CI)

### Risk of bias

The risk of bias across studies was considered high and the quality of evidence was rated very low in all analyses (Table 4). Being a complex intervention involving several variables, high-quality evidence is difficult to obtain [48]. Only one of the twenty-one studies that met the inclusion criteria was an RCT with possible high-quality evidence, stating no significant difference between mortality rates after PHI and EDI [29]. However, although the risk of bias in this study was low, it was not designed or powered to examine mortality as the primary outcome. The remaining 20 observational studies were all assessed as “fair” in our analysis. The rating of the quality of evidence from observational trials may be increased in some circumstances; due to possible confounding this was not achieved in any of our analyses [49]. A visual examination of the funnel plots did not reveal asymmetry consistent with publication bias (Additional file 4). Mortality was not uniformly reported across the studies; of the 21 included studies, 9 specified the survival to discharge, 2 reported 30-day mortality, and the remaining 10 reported “mortality” without any further description.

## Discussion

The aim of this review was to compare the mortality rates in adult trauma patients intubated before and immediately after hospital arrival. Despite differences between studies, our forest plots quite consistently showed a higher mortality rate for PHI than EDI. When all available data, both adjusted and unadjusted, were considered, no studies identified a positive effect on the mortality rate when PHI was compared to EDI. Eight separate analyses of subgroups were made, five of these found a significantly higher mortality rate in the PHI-patients; Crude mortality rate in both RSI (five studies) and non RSI (12 studies) studies, non RSI-studies after adjusting for injury severity (six studies), studies with no significant differences in ISS (four studies), and non RSI-studies with patients with a similar GCS (two studies). Three subgroup analyses did not identify a significant higher mortality rate after PHI; RSI-studies after adjusting for injury severity, based on one study, RSI-patients with a similar GCS, based on two studies, and studies from a European-organized EMS, based on two studies. However, there are some major objections towards doing a meta-analysis on this material: most importantly a high risk of selection bias and a high level of heterogeneity in the included studies.

The effect of selection bias in observational studies in this material should not be underestimated, as sicker patients are more prone to undergo more aggressive airway procedures. The fact that the only RCT included was also the only study with a non-significant trend towards a better mortality rate in the PHI group underlines this [29, 50]. We tried to weaken the impact of selection bias in this systematic review by only including studies with a high level of indication for intubation, reflected in all patients either undergoing PHI or EDI. Except for 2 studies, the articles examined in this review included only patients who had circulation at hospital admission, and patients who died shortly after hospital admission were excluded from the analysis in 11 of the 21 studies. In most of our included studies, the ISS in PHI patients was higher than in EDI patients. The lack of physiological parameters has been raised as an objection to the validity of the ISS when comparing patients, and a significantly higher mortality rate in the PHI group was shown in the four studies in which there were no differences between groups in the ISS [51]. The association between PHI and a higher mortality rate was similar for unadjusted and adjusted numbers, with an unadjusted OR of 2.54 (2.05, 3.15) and an adjusted OR of 2.59 (1.97, 3.39). The fact that the adjustments had little impact on the results is an interesting finding, which may imply that correcting for other factors associated with injury severity should be considered.

The other major factor in this meta-analysis is the high level of heterogeneity between the studies. Tracheal intubation (TI) is a complex intervention, patient populations are heterogeneous and there are major differences in staffing and EMS infrastructure. Only approximately 10% of the PHI patients in this meta-analysis had full access to RSI drugs; this reflects the clinical reality, but weakens the direct comparison of PHI to EDI. The subgroup analyses of studies where all PHI patients had access to RSI showed a less negative trend than for the studies in which RSI was not available for all, which suggests that access to pre-hospital RSI is of importance. One common objection to the comparison of PHI and EDI is that personnel outside the hospital, in general, receive less training in TI than their counterparts in the ED, which may lead to a prolonged performance time with increased exposure to hypoxia and possibly a higher rate of complications and failed intubation [12, 52–54]. Most studies in our analysis were from an American-organized EMS, in which paramedics perform most PHI; this differs from parts of Europe, where emergency physicians and anaesthesiologists perform most PHI (Table 1) [6]. Our subgroup analysis from a European-organized EMS was based on two studies and did not show a significant difference in mortality rates between PHI and EDI. A recent meta-analysis that examined success rates for PHI found a significantly higher median physician success rate of 98.8%, compared to a non-physician success rate of 91.7% (*p* = 0.003) [55]. The reported differences in success rates between PHI and EDI seem to be relatively low compared with the differences in mortality rates in our included studies, indicating that the differences in success rates alone may be insufficient to explain the observed differences in mortality rate. Success rate is, however, a very crude parameter with only two possible outcomes, and detailed information on time spent on the procedure, number of attempts before successful intubation, and adverse events that may influence patient status were not supplied in most studies. We aimed to examine subgroups of studies in which PHI was performed by personnel with the same level of expertise as those performing EDI, but the only study in which all patients were treated by physicians did not show any deviation from the other studies [43].

The high heterogeneity in this review is reflected in mortality rates of 7.7–93.5% for PHI and 6.25–66.5% for EDI, which gives an *I* ^2^ value of 91% in the crude data analysis (Fig. 2) and 86% in the adjusted OR analysis (Fig. 3). Any precise effect estimates or numbers needed to treat drawn from these heterogeneous data are necessarily invalid. One might argue that a meta-analysis of this material can be misleading and vague, but the high level of consistency present across a wide range of studies is still interesting. Despite the importance of selection bias and heterogeneity, to completely reject all negative results on grounds of methodology is not something that should be done without serious consideration, and a thorough investigation into other possible causes for differences in mortality rates seems to be strongly indicated.

Adverse events associated with TI are related not only to difficulty in inserting the airway but also the physiological consequences of the actual intubation and positive-pressure ventilation. The pre-hospital environment can be hostile, with few viable ways to treat complications. When muscle relaxants are administered, patients who previously had intact airway reflexes may face a greater risk of aspiration and hypoxia if difficulties occur. One study found transient hypoxia in more than half of the patients undergoing PHI RSI, which is significantly higher than the respective incidence for trauma intubations in the ED [52]. PHI may predispose to tension pneumothorax, and both the condition itself and therapeutic thoracotomy, if performed, have a relatively high morbidity rate [56]. Cardiovascular collapse is a known complication of TI in this patient group, and some centres deliberately postpone in-hospital TI in patients in shock until after initial stabilization [57, 58]. The only RCT included in our review identified a significantly higher occurrence of pre-hospital cardiac arrest after PHI; this may be related to Wang et al's finding of a  highly significant higher mortality rate after pre-hospital advanced airway management in patients with haemorrhagic shock, but no significantly higher mortality in patients without shock [59]. The studies in this review did not provide sufficiently detailed information to perform a separate analysis of patients in shock; this is a very important subgroup to investigate in future research into pre-hospital airway management.

None of the studies in this meta-analysis identified a significant positive effect on the mortality rate after PHI, but to interpret this as evidence that PHI is generally unfavourable does not seem to be valid. Many authors advocate the use of PHI, and the rationale for securing a seriously compromised airway as soon as possible seems reasonable, as the compromised patients are the same patients with the same problems, earlier in their pathway of care [18, 60]. It is unlikely that any pre-hospital services will achieve the level of care and equipment provided by a full in-hospital trauma team, which means that the rationale for PHI is that early protection and control of the airway outweighs the increased risks associated with performing the procedure in a less favourable setting. Regardless of the weaknesses concerning low-quality evidence, the consistent finding of worse outcomes after PHI compared with EDI should raise some questions. Variable effects in subgroups of patients have led to recommendations for a tailored approach to interventions in other fields of emergency care, and this may also be valid for pre-hospital airway management [61, 62].

## Conclusion

This systematic review quite consistently shows higher mortality rates when patients undergoing PHI are compared to patients intubated in the ED. However, reducing the analysis of a complex intervention to a dichotomous first-past-the-post approach discounts the comprehensive nature of the intervention. The association between PHI and a higher mortality rate does not necessarily contradict the importance of the intervention, but it does call for a thorough investigation by clinicians and researchers into possible causes for this finding. Further comparisons of widely defined patient and personnel groups are not likely to provide results that differ extensively from earlier reports; future research should include well-conducted subgroup analyses to investigate in which situations PHI may improve the outcome.

## Additional files

Additional file 1: Full search strategy. (DOCX 11 kb)

Additional file 2: Assessment table for observational trials. (DOCX 11 kb)

Additional file 3: Subgroup forest plots. (DOCX 2287 kb)

Additional file 4: Funnel plots. (DOCX 105 kb)

## Abbreviations

AOR: Adjusted odds ratio
CI: Confidence interval
ED: Emergency department
EDI: Emergency department intubation
EMS: Emergency medical services
GCS: Glasgow coma scale
GRADE: grading of recommendations assessment, development, and evaluation
ISS: Injury severity score
OR: Odds ratio
PHI: Pre-hospital intubation
RCT: Randomized controlled trial
RSI: Rapid sequence induction
TBI: Traumatic brain injury
TI: Tracheal intubation
