# Supplementary material for: A systematic review and meta-analysis comparing mortality in pre-hospital tracheal intubation to emergency department intubation in trauma patients
Source: Crit Care. 2017 Jul 31;21:192. doi: 10.1186/s13054-017-1787-x (PMC5535283; doi:10.1186/s13054-017-1787-x)
Supplement: Supplementary file 1 — Full search strategy. (DOCX 11 kb) [file 13054_2017_1787_MOESM1_ESM.docx]

Additional file 1, full search strategy:

OVID MEDLINE from 1946 to 2016 July 11:

Emergency Medical Service/(MeSH)

OR

prehospital*.mp.

OR

pre-hospital*.mp.

OR

out-of hospital*.mp.

OR

out of hospital*.mp.

AND

intubation, intratracheal (MeSH)

OR

airway management (MeSH)

OR

intubation*.mp.

OR

RSI*.mp.

(OVID EMBASE from 1974 to 05.12.13:

Keyword: emergency health service

OR

No related terms: Pre-hospital, Prehospital, out-of-hospital, out of hospital

AND

Keyword: intubation,

OR

Keyword: endotracheal intubation,

OR

RSI

OVID MEDLINE from 1946 to 05.12.13:

Emergency Medical Service/(MeSH)

OR

prehospital*.mp.

OR

Pre-hospital*.mp.

OR

out-of hospital*.mp.

OR

out of hospital*.mp.

AND

intubation, intratracheal (MESH)

OR

airway management (MeSH)

OR

Intubation*.mp.

OR

RSI*.mp.

The Cochrane Library: (Search all text was used for all searches)

Emergency Medical Service (MeSH)

OR

prehospital (word variations were searched)

OR

Pre-hospital (word variations were searched)

OR

out-of-hospital (word variations were searched)

OR

out of hospital (word variations were searched)

AND

intubation (MESH)

OR

airway management (MeSH)

OR

Intubation (word variations were searched)

OR

RSI
