# Supplementary material for: A systematic review and meta-analysis comparing mortality in pre-hospital tracheal intubation to emergency department intubation in trauma patients
Source: Crit Care. 2017 Jul 31;21:192. doi: 10.1186/s13054-017-1787-x (PMC5535283; doi:10.1186/s13054-017-1787-x)
Supplement: Supplementary file 2 — Assessment table for observational trials. (DOCX 11 kb) [file 13054_2017_1787_MOESM2_ESM.docx]

Additional file 2, assessment table for observational trials:

|  | Yes | No |
| --- | --- | --- |
| Clear definition of the study population |  |  |
| Clear definition of outcomes and outcome assessment for both patient groups |  |  |
| Directly comparable patient groups |  |  |
| Consistency in results |  |  |
| Important confounders and prognostic factors identified |  |  |
| Serious methodological limitations |  |  |
