# Supplementary material for: A systematic review and meta-analysis comparing mortality in pre-hospital tracheal intubation to emergency department intubation in trauma patients
Source: Crit Care. 2017 Jul 31;21:192. doi: 10.1186/s13054-017-1787-x (PMC5535283; doi:10.1186/s13054-017-1787-x)
Supplement: Supplementary file 3 — Subgroup forest plots. (DOCX 2287 kb) [file 13054_2017_1787_MOESM3_ESM.docx]

Additional file 3: Subgroup forest plots:


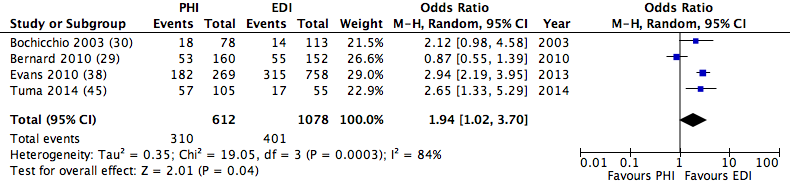


Forest plot 1: Studies with no significant differences in ISS between groups


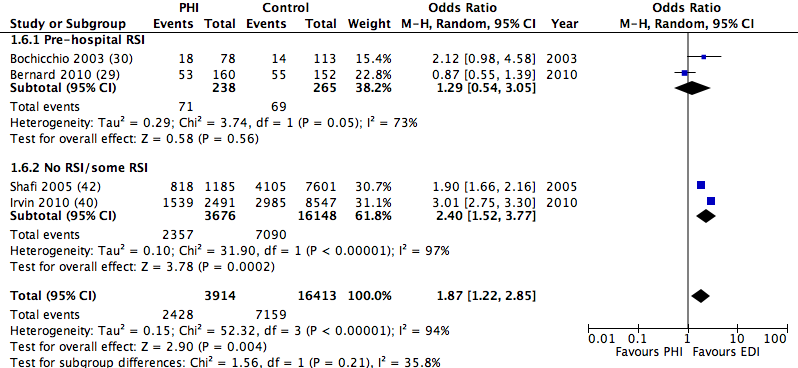


Forest plot 2: Studies with similar GCS of eight or less


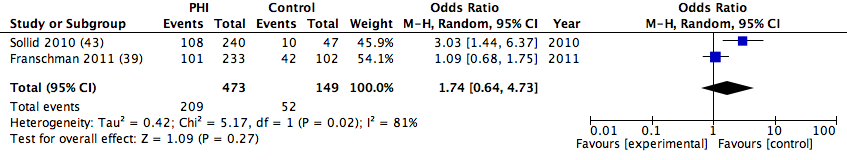


Forest plot 3: Studies from a European-organized EMS
