# Supplementary figures and images for: A systematic review and meta-analysis comparing mortality in pre-hospital tracheal intubation to emergency department intubation in trauma patients
Source: Crit Care. 2017 Jul 31;21:192. doi: 10.1186/s13054-017-1787-x (PMC5535283; doi:10.1186/s13054-017-1787-x)

Additional file 4, funnel plots:


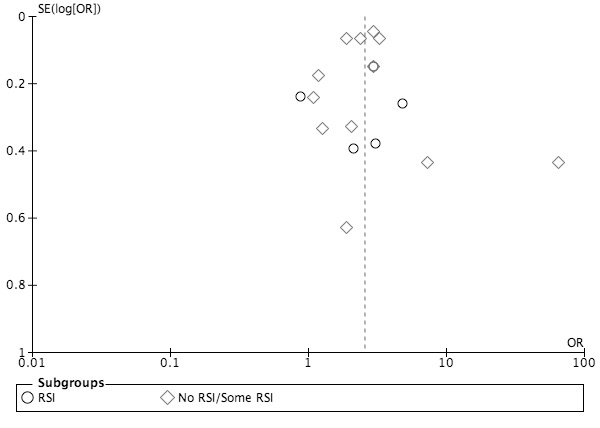


Funnel plot, crude data


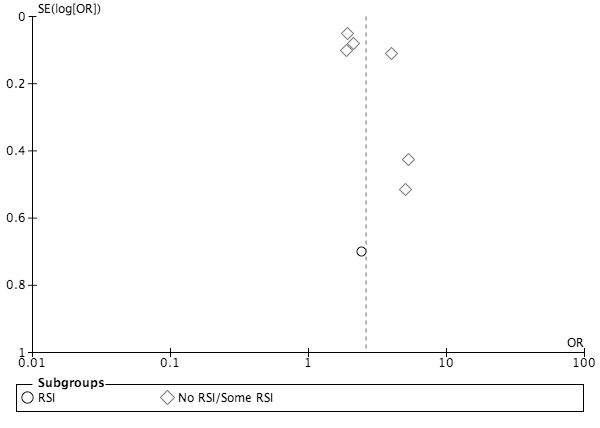


Funnel plot, adjusted data

Supplement: Supplementary file 4 — Funnel plots. (DOCX 105 kb) [file 13054_2017_1787_MOESM4_ESM.docx]
